# Brazilian Red Propolis Presents Promising Anti-*H. pylori* Activity in In Vitro and In Vivo Assays with the Ability to Modulate the Immune Response

**DOI:** 10.3390/molecules27217310

**Published:** 2022-10-27

**Authors:** Mariana B. Santiago, Luis Fernando Leandro, Rafael B. Rosa, Murilo V. Silva, Samuel C. Teixeira, João Paulo S. Servato, Sérgio Ricardo Ambrósio, Rodrigo Cassio S. Veneziani, Jennyfer A. Aldana-Mejía, Jairo K. Bastos, Carlos Henrique G. Martins

**Affiliations:** 1 Laboratory of Antimicrobial Testing, Institute of Biomedical Sciences, Federal University of Uberlândia, Uberlândia 38405318, MG, Brazil; 2 Complex of Animal Facilities, Federal University of Uberlândia, Uberlândia 38405315, MG, Brazil; 3 School of Dentistry, University of Uberaba, Uberaba 38055500, MG, Brazil; 4 Nucleus of Research in Sciences and Technolog, University of Franca, Franca 14404600, SP, Brazil; 5 Faculty of Pharmaceutical Sciences of Ribeirão Preto, University of São Paulo, Ribeirão Preto 14040900, SP, Brazil

**Keywords:** *Helicobacter pylori*, Brazilin red propolis, antibacterial, in vivo infection, synergism

## Abstract

*Helicobacter pylori* is a Gram-negative, microaerophilic, curved-rod, flagellated bacterium commonly found in the stomach mucosa and associated with different gastrointestinal diseases. With high levels of prevalence worldwide, it has developed resistance to the antibiotics used in its therapy. Brazilian red propolis has been studied due to its biological properties, and in the literature, it has shown promising antibacterial activities. The aim of this study was to evaluate anti-*H. pylori* from the crude hydroalcoholic extract of Brazilian red propolis (CHEBRP). For this, in vitro determination of the minimum inhibitory and bactericidal concentration (MIC/MBC) and synergistic activity and in vivo, microbiological, and histopathological analyses using Wistar rats were carried out using CHEBRP against *H. pylori* strains (ATCC 46523 and clinical isolate). CHEBRP presented MIC/MBC of 50 and 100 μg/mL against *H. pylori* strains (ATCC 43526 and clinical isolate, respectively) and tetracycline MIC/MBC of 0.74 µg/mL. The association of CHEBRP with tetracycline had an indifferent effect. In the stomach mucosa of rats, all treatments performed significantly decreased the number of *H. pylori*, and a concentration of 300 mg/kg was able to modulate the inflammatory response in the tissue. Therefore, CHEBRP showed promising anti-*H. pylori* in in vitro and in vivo assays.

## 1. Introduction

*Helicobacter pylori* is a Gram-negative, microaerophilic, spiral, S-shaped/curved-rod bacterium measuring about 3 to 5 μm in length by 0.5 μm in width, with a smooth outer cell wall and four to six sheathed unipolar flagella with a terminal bulb [1]. Prevalence across the world varies; however, it is known that the bacterium colonizes the gastrointestinal mucosa, the stomach antrum being its main site, of almost half the global human population [2,3]. It is believed that *H. pylori* infection occurs in childhood, where the oral-oral and oral–fecal routes are considered possible forms of transmission. When colonized by the bacterium, the child becomes a healthy carrier, and may or may not have symptoms of *H. pylori* infection during adulthood [2,4]. It has virulence and adaptation factors that are associated with the development of different gastrointestinal diseases (e.g., gastric and duodenal ulcers and gastric cancer) [3,4,5].

The gastric mucosa is considered an extremely hostile environment, mainly due to the high concentration of hydrochloric acid; however, *H. pylori* has mechanisms that allow it to overcome barriers and colonize, and survive, in the site. Upon reaching the stomach, *H. pylori*, through its urease activity, neutralizes the acidic environment of the organ. Due to the presence of its flagella, the bacteria have motility and are thus is able to move until reaching the gastric epithelium, where the bacterial adhesins interact with the host’s receptor cells, causing the colonization of *H. pylori*. After colonization is completed, bacteria begin to replicate and cause damage to the host tissue through the various toxins and effector proteins produced. The clinical manifestation of the infection occurs through the activation of innate immunity, triggered by the secretion of chemokines by the bacteria and activation of neutrophils [4].

Infection caused by *H. pylori* activates complex host immune responses, involving both innate and adaptive response mechanisms. Upon contact with bacteria, *H. pylori* antigens bind to receptors on gastric cells, activating nuclear factor kappa B (NF-κB) and c-Jun N-terminal kinase (CNK) pathways, followed by the release of proinflammatory cytokines [6]. Next, neutrophils and mononuclear cells infiltrate the gastric mucosa, resulting in the production of nitric oxide. Adaptive immune cells, CD4+ T lymphocytes, and CD8+ T lymphocytes are also recruited. Furthermore, the cytokine profile in patients with *H. pylori* infection, high levels of gamma interferon, tumor necrosis factor and interleukins, in addition to having proinflammatory effects during infection, suggest that the host response follows a Th1-polarized response [6].

Protocols for the treatment of *H. pylori* infection recommend that it be performed with first-line triple therapy, using a proton pump inhibitor (e.g., omeprazole) and two antibiotics (e.g., clarithromycin, tetracycline, metronidazole and amoxicillin). However, the increase in bacterial resistance to antibiotics, such as clarithromycin, has been alarming and the main factor for therapeutic failure [7]. The concern is such that in 2017 the World Health Organization (WHO) published a list containing 12 antibiotic-resistant bacteria that pose a threat to human health, and in this list the development of new antibiotics against clarithromycin-resistant *H. pylori* was classified as high priority [8]. Another problem is that the high dose during a long administration frequency and long duration of treatment is responsible for causing several side effects (e.g., nausea, vomiting, diarrhea and dizziness), accountable for poor patient compliance [7].

In this sense, natural products are sources of active substances that can be used therapeutically due to the great diversity of metabolites produced [9]. The use of these resources for medicinal purposes, whether in treatment, cure or prevention of pathologies, is one of the oldest forms of medicinal practice of humanity [10]. Brazil is the most biodiverse country in the world and many studies are carried out seeking to evaluate the biological properties of natural products, and their compounds, found in the country, in order to develop therapeutic alternatives against diseases [11].

Several natural products with anti-*H. pylori* properties are found in the scientific literature, such as *Aloe vera* [12], *Syzygium aromaticum* [13], *Canarium album* Raeusch. [14], essential oils and South African honey [15]. Due to the scientific interest in finding therapeutic alternatives in natural products to control and combat the infection of *H. pylori*, and the good results found, it is promising to look for new products that have not yet been studied that may present this property.

Propolis, popularly known as “bee glue,” is a product produced by bees from buds, flowers and different plant exudates, to which bees add salivary secretions, wax and pollen for the final preparation of the substance [16]. In recent years, the scientific literature on propolis has been reporting promising pharmacological properties, where great antimicrobial potential can be highlighted [17,18]. The chemical compounds of natural products are heterogeneous and may vary according to their geographical location and the time of harvest [19,20]. Propolis is mainly composed of resin (50%), wax (30%), essential oils (10%), pollen (5%) and organic compounds (5%). Flavonoids, phenolic compounds, esters, alcohols, terpenes and beta-steroids can be mentioned as important organic compounds already identified in propolis samples [21]. To date, the scientific literature has reported the identification of about 300 different chemical components in propolis from different sources [22].

Brazilian red propolis is a product of the *Apis mellifera* bee due to the collection of red exudates on the surface of the trunks of *Dalbergia ecastophyllum*, popularly known in Brazil as *rabo de bugio*, which is the botanical origin of red propolis [23,24,25]. The Brazilian red propolis presents in its chemical composition compounds that are not found in propolis samples from other sources, due to the vast Brazilian biodiversity and its botanical source. Brazilian red propolis has bioactivities reported in the literature, such as antioxidant, antibacterial, antiviral, antiparasitic, anti-inflammatory and wound healing, antitumor and antiproliferative activity, and immunomodulatory action, among others [25].

To the best of our knowledge, only one study in the literature has reported the in vitro anti-*H. pylori* activity of the Brazilian red propolis [26]. For this purpose, this study evaluated the in vitro and in vivo antibacterial activity of the crude hydroalcoholic extract of Brazilian red propolis (CHEBRP) against strains of *H. pylori*.

## 2. Results

### 2.1. CHEBRP Chemical Characterization

**Figure 1:**
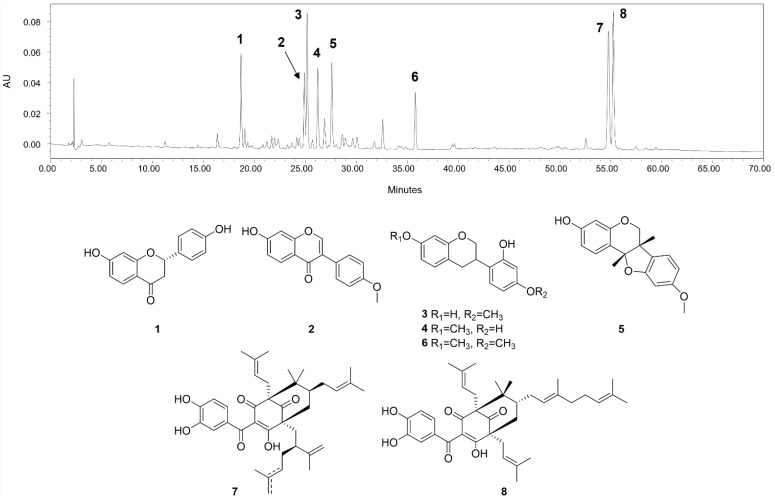
HPLC chromatographic profile (275 nm) and chemical structures of the main compounds identified in CHEBRP. Numbers correspond to liquiritigenin (**1**), formononetin (**2**), vestitol (**3**), neovestitol (**4**), medicarpin (**5**), 7-O-neovestitol (**6**), guttiferone E (**7**), and oblongifolin B (**8**).

The main compounds identified in CHEBRP are liquiritigenin, formononetin, vestitol, neovestitol, medicarpin, 7-O-methylvestitol, a mixture of guttiferone E and xanthochymol, and oblongifolin B (Figure 1).

### 2.2. In Vitro Evaluation

In vitro evaluation of anti-*H. pylori* activity of CHEBRP was performed by determining the minimal inhibitory concentration (MIC), minimal bacterial concentration (MBC), and synergistic activity.

The determination of the MIC of CHEBRP obtained results of 50.0 and 100.0 μg/mL against strains *H. pylori* (ATCC 43526) and *H. pylori* (clinical isolate), respectively. The determination of MBC obtained the same results (as found in the MIC) against the same strains. Therefore, the bactericidal effect of CHEBRP against the evaluated bacteria was determined (Table 1). Tetracycline was used as a positive control in both assays, and its MIC and MBC were determined to be 0.74 μg/mL against both strains evaluated in the present study (Table 1).

The synergistic activity of CHEBRP in combination with the antibiotic tetracycline was evaluated using the fractional inhibitory concentration index (FICI). The combination of the two substances against the strains of *H. pylori* had an indifferent effect (Table 2).

### 2.3. In Vivo Evaluation

For the in vivo evaluation of the anti-*H. pylori* activity of CHEBRP, the assays were performed using Wistar rats as animal models. Through microbiological and histopathological analysis, a quantitative and qualitative evaluation of the effect of defined concentrations of CHEBRP in the gastric tissue (antrum) of the rats was carried out.

**Figure 2:**
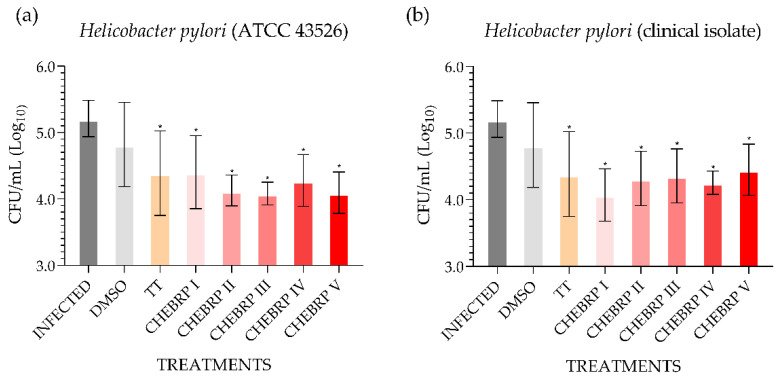
Quantitative determination of the presence of *Helicobacter pylori* in the stomach mucosa (antrum) of Wistar rats. DMSO—dimethyl sulfoxide. TT—triple therapy (amoxicillin 50.0 mg/kg, clarithromycin 25.0 mg/kg, and omeprazole 20.0 mg/kg). CHEBRP—concentrations of crude hydroalcoholic extract of Brazilian red propolis (I: 18.25 mg/kg; II: 37.5 mg/kg; III: 75.0 mg/kg; IV: 150.0 mg/kg and V: 300.0 mg/kg). (**a**) Determination of the quantity of *Helicobacter pylori* (ATCC 43526) in the gastric antrum after treatments. (**b**) Determination of the quantity of *Helicobacter pylori* (clinical isolate) in the gastric antrum after treatments. * *p* < 0.05 compared to control (infected).

The microbiological analysis performed using the technique of counting colony-forming units (CFU)/mL determined that all treatments used, except the one performed with dimethyl sulfoxide (DMSO), for both strains evaluated statistically decreased the quantity of bacteria in the antrum of rats (Figure 2).

Histopathological analyses were performed to qualitatively evaluate the inflammatory infiltrate, activity, epithelial atrophy and presence of metaplasia. In the evaluation of the histological sections, it was determined that CHEPBR at a concentration of 300.0 mg/kg and triple therapy (TT: amoxicillin 50.0 mg/kg, clarithromycin 25.0 mg/kg, and omeprazole 20.0 mg/kg) were the only treatments performed that were capable of decreasing the density of *H. pylori* (ATCC 43526 and clinical isolate) and modulating the inflammatory response (Table 3 and Table 4).

**Figure 3:**
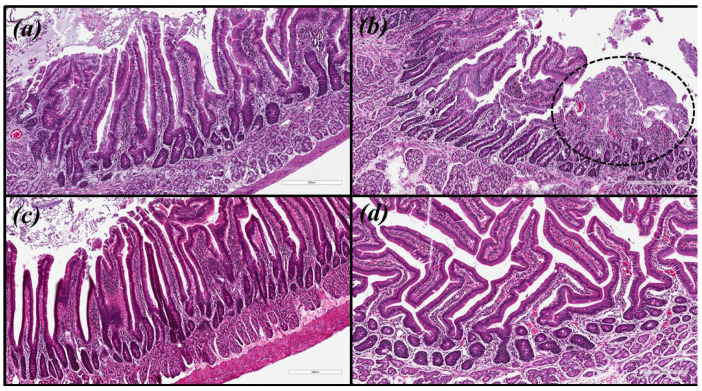
Biological tissue images (antrum-pyloric) of rats stained with hematoxylin and eosin (HE). (**a**) Uninfected group. (**b**) Infected group—the circled area demonstrates the ulceration and the intense mononuclear inflammatory infiltrate in the submucosa. (**c**) Group infected and treated with triple therapy (amoxicillin 50.0 mg/kg, clarithromycin 25.0 mg/kg, and omeprazole 20.0 mg/kg)—note the integrity of the epithelial lining and the lower density of lymphocytic infiltrate in the submucosa. (**d**) Group infected and treated with 300 mg/kg of crude hydroalcoholic extract of Brazilian red propolis—note the integrity of the epithelial lining and the lower density of lymphocytic infiltrate in the submucosa.

**Figure 4:**
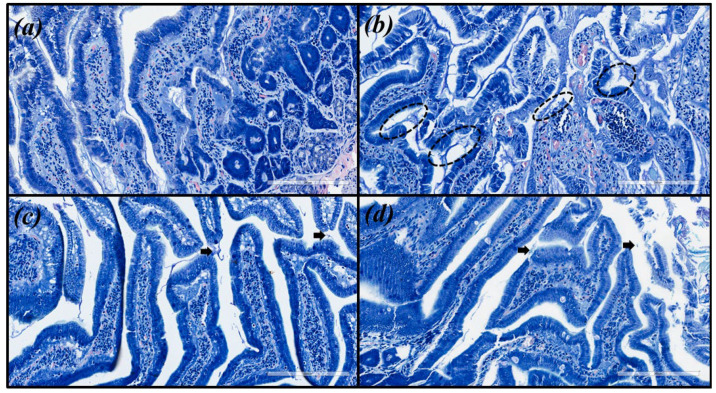
Images of the biological tissue (antrum-pyloric) of rats stained with modified Giemsa. (**a**) Uninfected group. (**b**) Infected group—black circles indicate stained *H. pylori* (blue) that are attached to the gastric epithelial cells (moderated density). (**c**) Group infected and treated with triple therapy (amoxicillin 50.0 mg/kg, clarithromycin 25.0 mg/kg, and omeprazole 20.0 mg/kg)—the arrows point to occasionally stained *H. pylori* (mild density). (**d**) Group infected and treated with 300 mg/kg of crude hydroalcoholic extract of Brazilian red propolis—the arrow point to occasionally stained *H. pylori* (mild density).

Microscopic images were obtained for hematoxylin and eosin (HE) and with modified Giemsa staining (Figure 3 and Figure 4, respectively). The same pattern of inflammation and bacterial infection was observed (both strains analyzed) for the untreated and those treated with CHEBRP at concentrations of 18.25, 37.5, 75.0 and 150.0 mg/kg. The same pattern was also observed for the groups treated with TT and CHEBRP at 300.0 mg/kg.

These last two treatments were the only ones capable, according to the updated Sydney classification [27,28], of decreasing chronic inflammation from moderate to mild, neutrophil polymorph infiltration (activity) from mild to absent, macrophages from mild to absent, tissue damage from mild to absent, apoptotic bodies from mild to absent, lamina propria edema from moderate to absent, epithelial/glandular atrophy from mild to absent, intestinal metaplasia from mild to absent, and bacterial (*H. pylori* ATCC 43526 and clinical isolate) density from moderate to mild.

## 3. Discussion

In the scientific literature, no other studies were found in which the anti-*H. pylori* activity of CHEBRP was determined. Therefore, the results found in this study, in which these properties of CHEBRP were evaluated by in vitro and in vivo analyses, are unprecedented.

In the present study, CHEBRP presented an MIC value of 50.0 and 100.0 μg/mL against *H. pylori* (ATCC 43526) and *H. pylori* (clinical isolate), respectively. In their study, Holetz et al. [29] considered good antibacterial activity when extracts have an MIC value lower than 100.0 μg/mL, moderate activity when MIC is from 100.0 to 500.0 μg/mL, weak activity when the concentration varies from 500.0 to 1000 μg/mL, and inactive when it is over 1000 μg/mL. Considering the same criteria used by the authors [29] and the results obtained in the present study, CHEBRP showed promising anti-*H. pylori* activity, since, against *H. pylori* (ATCC 43526) the activity was good and against *H. pylori* (clinical isolate) the activity was moderate.

Baltas et al. [30] evaluated the antimicrobial activity of 15 samples of propolis, acquired from different areas of Turkey, against the strain *H. pylori* J99 through the agar-well diffusion assay. At a concentration of 75.0 μg/mL, the zones of inhibition ranged from 31 to 47 mm. Boyanova et al. [31] evaluated the ethanolic extract of Bulgarian propolis against 94 strains of *H. pylori* (clinical isolate), also through the agar-well diffusion assay, at concentrations of 9000, 18,000 and 27,000 μg per well, and found zones of inhibition that ranged from 7 to 48, 7 to 56.5, and 9 to 60 mm, respectively. Villanueva et al. [32], also chose concentrations of 9000, 18,000 and 27,000 μg per well to evaluate the antibacterial activity of 22 Chilean propolis extracts against 10 strains of *H. pylori* through the agar-well diffusion assay. The authors determined zones of inhibition that ranged from 35 to 77, 31 to 60 and 26 to 54 mm in wells containing 27,000, 18,000 and 9000 μg/well of propolis, respectively. Mendonça et al. [26] evaluated the Brazilian red propolis extract, also through the agar-well diffusion method, and determined that 80 μL of Brazilian red propolis at a concentration of 100,000 μg/mL causes 13.0 ± 2.0 mm inhibition of *H. pylori* (ATCC 43504) The present study used the broth dilution assay to determine the MIC of CHEBRP against strains of *H. pylori*, due to the difference in methodology used, it is not possible to compare the results obtained in this study with those obtained by the authors described above. However, it is possible to verify that the propolis extract from different regions has good antibacterial activity against *H. pylori*, which, with further studies, could become a therapeutic alternative.

Widelski et al. [33] determined the MIC/MBC of propolis extract samples—four from Poland, four from Ukraine, one from Kazakhstan, and one from Greece—against *H. pylori* (ATCC 43504). The results found ranged from 15.0 to 60.0 μg/mL. In the present study, against *H. pylori* (ATCC 43526), CHEBRP presented an MIC/CBM of 50.0 μg/mL, a lower result than that found by Widelski et al. [33] from a propolis sample collected from Poland against *H. pylori* (ATCC 43504), which obtained an MIC/MBC of 60.0 μg/mL. The other nine extracts evaluated by the authors obtained MBC of 30 μg/mL against this strain. In the present study, the CBM concentration of CHEBRP was only 20 μg/mL higher against *H. pylori* (ATCC 43526). These results suggest that the data obtained in this present study are within the expected range, since they are in agreement with the observations of previous studies.

In previous studies, our research group evaluated the MIC and MBC of the hydroalcoholic crude extract from the leaves of nine *Copaifera* spp. against *H. pylori* (ATCC 43526). MIC and MBC were determined for: *C. trapezifolia* (12.5/25 μg/mL), *C. duckei* (12.5/12.5 μg/mL), *C. oblongifolia* (50.0/50.0 μg/mL), *C. langsdorffii* (25.0/100.0 μg/mL), *C. pubiflora* (100.0/100.0 μg/mL), *C. paupera* (50.0/50.0 μg/mL), *C. reticulata* (50.0/50.0 μg/mL), *C. multijuga* (25.0/25.0 μg/mL) and *C. lucens* (25.0/25.0 μg/mL) [34]. In the present study, against the same strain, CHEBRP presented MIC/MBC of 50.0 μg/mL, the same concentration as previously obtained for *C. oblongifolia*, *C. paupera* and *C. reticulta*, and lower than that obtained for *C. langsdorffii* and *C. pubiflora* [34]. These results underline the fact that natural products have strong anti-*H. pylori* activities and can contribute to fighting bacterial infection.

As the fight against bacterial infection is carried out through therapies that use a combination of antibiotics [7], it is interesting that any proposed therapeutic novelty evaluates the synergistic action of substances with standard antibiotics. In this sense, Nostro et al. [35] evaluated the synergistic activity of commercial propolis resin (Italy) in combination with clarithromycin against 25 strains of *H. pylori* (24 clinical isolates and one ATCC 43504), of which nine showed synergism, nine antagonism, and seven indifferent. In the present study, the combination of CHEBRP with tetracycline presented an indifferent result against the strains evaluated (*H. pylori* ATCC 43526 and clinical isolate).

The CHEBRP used in this study has among its compounds liquiritigenin, isoliquiritigenin, formononetin, vestitol, neovestitol, medicarpin, 7-O-methylvestitol, guttiferone E/xanthochymol, and oblongifolin B. Studies suggest that compounds such as medicarpin, vestitol and neovestitol commonly found in Brazilian red propolis samples and found in the CHEBRP used in the present study may be responsible for the antibacterial action of Brazilian red propolis [36,37]. However, studies also indicate that a synergistic effect between its compounds is related to biological properties presented by CHEBRP [38,39].

Fukai et al. [40] evaluated the anti-*H. pylori* activity of the compounds formononetin and vestitol, and it was determined that formononetin showed weak anti-*H. pylori* activity (MIC > 100 μg/mL) and vestitol good antibacterial activity (MIC 12.5 μg/mL) against *H. pylori* (ATCC 43526). The other six main compounds present in CHEBRP (liquiritigenin, isoliquiritigenin, neovestitol, medicarpin, 7-O-methylvestitol, guttiferone E/xanthochymol, and oblongifolin B) have not yet been reported in the literature to have anti-*H. pylori* activity. In the gastroprotective evaluation of compounds isolated from Brazilian red propolis in mice with ethanol-induced ulcers, Boing et al. [38] determined that the compound medicarpin is essential for the extract to present this biological property.

Due to promising anti-*H. pylori* activity in vitro from Brazilian red propolis in the present study, and the fact that the extract showed gastroprotective properties in previous studies [38,41], supports the realization of the anti-*H. pylori* activity in vivo performed.

Mendonça et al. [26] evaluated the gastroprotective activity of Brazilian red propolis, collected in Sergipe and its major compound being formononetin, in the reduction of gastric ulceration caused by indomethacin (100.0 mg/kg) in Wistar rats. The Brazilian red propolis used by the authors was able to reduce gastric ulceration by 87.34% and 100.0% with doses of 250.0 and 500.0 mg/kg. The CHEBRP used in the present study was collected in Bahia, and its components differ from the Brazilian red propolis used by Mendonça et al. [26] due to the geographical difference in propolis collection [22]. However, both the Brazilian red propolis, the one used in the present study and the one used by Mendonça et al. [26] showed gastroprotective action in in vivo assays using Wistar rats. Therefore, the data obtained in the present study are in agreement with the scientific literature.

In order to develop a vaccine against *H. pylori*, Mahboubi et al. [42] evaluated in C57BL/6 mice the action of Iranian propolis alone and in combination with outer inflammatory protein A (OipA), and OipA alone against *H. pylori* (clinical isolate). The concentration used by the authors of propolis was 10.0 mg/dose and of OipA 100.0 μg/dose. All vaccines used by the authors decreased the concentration of *H. pylori* (CFU/g) in the stomach of infected mice significantly compared to the unvaccinated control. However, when propolis was combined with OipA, the results were significantly greater than for propolis and OipA alone, and yet the effect of OipA was significantly smaller than for propolis, suggesting that some compound of Iranian propolis was antagonistic to OipA [42]. In the present study, in Wistar rats, concentrations of CHEBRP (18.25, 37.5, 75.0, 150.0 and 300.0 mg/kg) were used, and all concentrations of CHEBRP were able to significantly decrease the quantity of *H. pylori* (ATCC 43526 and clinical isolate) in the stomach (antrum) of rats when compared to the untreated control, corroborating earlier findings.

A clinical study evaluated the action of Brazilian green propolis in 18 patients who had confirmed *H. pylori* infection [43]. Participants received 20 drops of green propolis three times a day for seven days. However, it did not present promising results, which the authors attributed to the treatment with low dosage of Brazilian green propolis during a short time of administration [43]. The same hypothesis can be raised in relation to the CHEBRP used in the present study, even though, all the concentrations evaluated, having significantly reduced the quantity of *H. pylori* in the antrum of the rats, did not completely eliminate the bacterial load in the tissue. More studies are needed to assess whether there is any dose–time relationship that influences anti-*H. pylori* in vivo activity of CHEBRP.

Many studies are still needed to better evaluate the biological properties of CHEBRP before it can be considered a therapeutic alternative against infection caused by *H. pylori*. However, the data obtained in the present study are a great advance on the way to better knowledge about the extract, since they are unprecedented.

## 4. Materials and Methods

### 4.1. Crude Hydroalcoholic Extract of Brazilian Red Propolis

CHEBRP (submitted to the National System for the Management of Genetic Heritage and Associated Traditional Knowledge—SISGEN, registered as AF234D8) was obtained from the Association of Beekeepers of Canavieiras (Cooperativa de Apicultores de Canavieiras, COAPER, Bahia, Brazil), a Brazilian municipality located in the southern region of Bahia, northeastern Brazil in March 2019. For extraction, Brazilian red propolis samples were frozen and minced. Two hundred grams of Brazilian red propolis were submitted to dynamic maceration at 30 °C and 120 rpm using a shaker incubator (INNOVA 4300, New Brunswick Scientific, Saint Albans, UK) with 70% hydroalcoholic ethanol solution. The extracts were concentrated under vacuum using a rotary evaporator and lyophilized to complete dryness.

The phytochemical characterization of CHEBRP was performed by high-performance liquid chromatography with a photodiode array detector (HPLC-PAD) on a Waters 1500 series setup (Milford, CT, USA). A Synergy Fusion RP-80 A column (150 × 4.6 mm, 4 μ; Phenomenex, Torrance, CA, USA) was used as a stationary phase, and water with 0.1% formic acid (A) and acetonitrile (B) as the mobile phase. The injections were performed on a gradient elution method of 10–100% of B (60 min), 100% of B (70 min), with a flow rate of 1 mL/min, at 40 °C.

Previously authentic standards from CHEBRP isolated by Aldana-Mejia et al. [44] were used to identify the major compounds on the sample. The standard compounds included liquiritigenin, formononetin, vestitol, neovestitol, medicarpin, 7-O-methylvestitol, guttiferone E/xanthochymol, and oblongifolin B. The purity of the standards was estimated to be greater than 96% by HPLC and NMR [44].

### 4.2. Bacterial Strains Used in the Assays

All strains used are part of the culture collection of the Laboratory of Antimicrobial Testing (LEA) of the Federal University of Uberlândia (UFU), and are kept under cryopreservation (−80 °C) in Brucella broth (Difco Labs, Detroit, MI, USA) supplemented with 10.0% fetal bovine serum. One strain comes from the American Type Culture Collection (ATCC 43526), and a clinical isolate was isolated from clinical trials, in a previous study, from a patient with peptic ulcer caused by bacterial infection (clinical isolate).

### 4.3. Determination of Minimal Inhibitory and Bactericidal Concentrations

The MIC is defined by the lowest concentration of the antimicrobial agent capable of inhibiting bacterial growth. For its determination, the microdilution method was carried out in a 96 well microplate, according to the methodology recommended by the Clinical and Laboratory Standards Institute (CLSI) [45], with adaptations, using resazurin (Sigma, St. Louis, MO, USA) as bacterial growth developer [46] and Brucella broth (Difco) supplemented with 10% fetal bovine serum.

Briefly, CHEBRP was dissolved in 5% DMSO to reach final concentrations ranging from 0.195 to 400.0 μg/mL. The bacterial inoculum was adjusted to produce a cell concentration equal to 5 × 10^5^ CFU/mL. Tetracycline (Sigma) in concentrations ranging from 0.0115 to 5.9 μg/mL was used as a standard antibiotic and solubilized in water. Control of 5% DMSO was performed, and the solvent did not interfere with bacterial growth at this concentration. The plates were incubated in a CO_2_ incubator (Panasonic Biomedical, Netherlands, Amsterdam) at 37 °C for 72 h in the atmosphere containing 10% CO_2_. After the incubation period, 30.0 μL of a 0.01% aqueous resazurin solution was added to each well of the microplate to assess bacterial growth [46].

The MBC, defined as the lowest concentration capable of completely preventing bacterial growth, was performed in the MIC microplate, prior to the addition of resazurin. An aliquot of 10.0 μL was taken from all microplate wells, and inoculated onto Brucella agar (Difco) supplemented with 5.0% defibrinated horse blood and 10.0% fetal bovine serum. Then, the agar plates were incubated under the same conditions and time as the microplates described above. After the incubation period, it was found at the lowest concentration that no bacterial growth occurred. All assays were performed in triplicate.

### 4.4. Determination of Synergistic Activity

Determination of synergistic activity was performed according to the protocol described by Chaturvedi et al. [47]. Concentrations of CHEBRP and tetracycline (Sigma) were combined in the standard MIC format against 5 × 10^5^ CFU/mL of bacterial inoculum. To assess synergism, fractional inhibitory concentration index (FICI) values were calculated as previously established in the literature [47]. The index values were analyzed as follows: ≤0.5, synergism; >0.5 to <1.0, additive; ≥1.0 to <4.0, indifference; and ≥4.0, antagonism [48]. The assay was performed in triplicate.

### 4.5. Determination of Anti-H. pylori Activity in Wistar Rats

For in vivo assessment of anti-*H. pylori* from CHEBRP against the two bacterial strains used, the method developed by Werawatganon [49], with adaptations, was performed. The evaluation was approved by the Ethics Committee on the Use of Animals of the Federal University of Uberlândia (Protocol n. 095/2019).

Briefly, 126 male Wistar rats weighing an average of 300 to 350 g were used in the trial. Initially, the animals were randomly divided into groups according to the treatment, *H. pylori* infection was induced in the animals through gavage with a bacterial inoculum adjusted to a coresponder from 10^8^ to 10^9^ CFU/mL for three days twice a day. After confirming the infection, performed by the OnSite *H. pylori* Ag rapid test (CTK Biotech, Inc. San Diego, CA, USA) in the animals’ feces, the treatment was performed, also via gavage, with concentrations of CHEBRP (18,25, 37.5, 75.0, 150.0 and 300.0 mg/kg) solubilized in 1% DMSO and triple therapy (TT—amoxicillin 50.0 mg/kg, clarithromycin 25.0 mg/kg, and omeprazole 20.0 mg/kg) solubilized in water for seven days once a day. Each treatment group had seven rats and the necessary controls were also performed (inoculated, non-inoculated and solvent). The maximum volume of gavage received was 0.8 milliliters per rats.

After the treatment period, the animals were euthanized in a CO_2_ chamber and the biological material of study (stomach) was collected, the stomach antrum was processed and divided into two parts, one for microbiological analysis (quantitative) and another for histopathological analysis (qualitative).

The microbiological analysis was performed through counting that determined the number of CFU/mL in the stomach tissue. The material was placed in Brucella broth (Difico) supplemented with 10.0% fetal bovine serum and vortexed for one minute. Afterwards, dilutions ranging from 10^0^ to 10^7^ were performed, and seeded on Brucella agar (Difico) supplemented with 5.0% horse blood and 10.0% fetal bovine serum, containing vancomycin (Sigma—5.0 mg), polymyxin B (Sigma—2.5.0 mg), and trimethoprim (Sigma—2.5 mg) to suppress the growth of other bacteria and fungi [50]. The plates were incubated at 37 °C for 72 h in a 10.0% CO_2_ incubator. After this period, the count was performed which determined CFU/mL in the tissue.

For histopathological analysis, gastric tissue samples were fixed in 10.0% buffered formalin solution, dehydrated in ethanol solution, cleared in xylene and embedded in paraffin. Blocks containing hearts were sectioned into 5.0 μm-thick sections and then placed on glass slides and routinely stained with hematoxylin and eosin (HE) and modified Giemsa stain [51].

To assess inflammatory infiltrate, activity, epithelial atrophy, and presence of metaplasia, slides were stained with hematoxylin and eosin (HE). The density of *H. pylori* was evaluated in sections stained with modified Giemsa. The type and severity of the inflammatory infiltrate and tissue damage were determined by two independent observers who were calibrated for the presence or absence of (i) inflammatory response, (ii) neutrophils, (iii) macrophages, (iv) lymphocytes, (v) plasma cells, (vi) foreign body giant cells, (vii) tissue damage, (viii) necrotic tissue, (ix) apoptotic bodies, (x) edema, (xi) epithelial atrophy, (xii) intestinal metaplasia, (xiii) density of *H. pylori*. These characteristics were scored for intensity: (-) absent, (+) mild, (++) moderate, (+++) severe, as previously described by the updated Sydney classification [27,28].

### 4.6. Statistical Analysis

The data obtained in the microbiological evaluation (quantitative) were statistically analyzed by analysis of variance (ANOVA) using GraphPad Prism version 8.0.2 for Windows (GraphPad Software, San Diego, CA, USA), with calculation of the F statistic and its respective *p*-value. In cases where *p* < 0.05, treatment means (Log_10_) were compared using Dunnett’s multiple comparisons test, with the calculation of the minimum significant difference for α = 0.05.

## 5. Conclusions

Based on the data obtained in the present study, it can be concluded that the crude hydroalcoholic extract of red propolis has promising anti-*H. pylori* activity in vitro, due to its bactericidal capacity, at low concentrations, against standard and clinically isolated strains and not interfering with the effect of an antibiotic used in standard therapy. CHEBRP also shows promising anti-*H. pylori* activity in vivo, since the microbiological and histopathological studies performed showed the extract’s ability to reduce bacterial load and modulate the immune response in the biological tissue of mice infected by the strains evaluated. However, more studies are needed to better analyze this biological property (e.g., antibiofilm activity, mechanisms of action), and our research group intends to continue this investigation.

## Figures and Tables

**Table 1:** Determination of Minimal Inhibitory and Bactericidal Concentrations of the crude hydroalcoholic extract of Brazilian red propolis against *Helicobacter pylori* strains (ATCC 43526 and clinical isolate).

Strains	CHEBRP		Tetracycline	
	MIC	MBC	MIC	MBC
	(μg/mL)			
*Helicobacter pylori* (ATCC 43526)	50.0	50.0	0.74	0.74
*Helicobacter pylori* (clinical isolate)	100.0	100.0	0.74	0.74

CHEBRP—crude hydroalcoholic extract of Brazilian red propolis. MIC—minimal inhibitory concentration. MBC—minimal bacterial concentration.

**Table 2:** Determination of the synergistic activity of the hydroalcoholic extract of Brazilian red propolis in combination with tetracycline against *Helicobacter pylori* strains (ATCC 43526 and clinical isolate).

Strains	MICa (μg/mL)		MICb (μg/mL)		FIC *		FICI **	Outcome ***
	(1)	(2)	(1)	(2)	(1)	(2)		
*Helicobacter pylori* (ATCC 43526)	50.0	1.48	50.0	0.74	1.0	0.5	1.5	Indifferent
*Helicobacter pylori* (clinical isolate)	200.0	1.48	200.0	1.48	1.0	1.0	2.0	Indifferent

MICa—minimal inhibitory concentration alone. MICb—combined minimum inhibitory concentration. FIC—fractional inhibitory concentration. FICI—fractional inhibitory concentration index. (1)—Hydroalcoholic crude extract of Brazilian red propolis. (2)—Tetracycline. * FIC = MICb/MICa. ** FICI = FIC (1) + FIC (2). *** Synergistic interaction: FICI ≤ 0.5. Additive: FICI > 0.5 and <1.0. Indifferent: FICI > 1.0 and <4.0. Antagonistic interaction: FICI ≥ 4.0.

**Table 3:** Qualitative analyses of gastric tissue (antrum-pyloric) of the experimental groups during *Helicobacter pylori* (ATCC 43526) infection in rats.

Histological Criteria	Uninfected	Infected	DMSO^¤^	TT^¤^	CHEBRP I^¤^	CHEBRP II^¤^	CHEBRP III^¤^	CHEBRP IV^¤^	CHEBRP V^¤^
Chronic inflammation	+ **	++	++	+	++	++	++	++	+
Neutrophil polymorph infiltration (activity)	-	+	+	-	+	+	+	+	-
Eosinophils	-	-	-	-	-	-	-	-	-
Macrophages	-	+	+	-	+	+	+	+	-
Lymphocytes	+	++	++	++	++	++	++	++	++
Plasma cells	+	+	+	+	+	+	+	+	+
Giant foreign body cells	-	-	-	-	-	-	-	-	-
Fibroblast	-	-	-	-	-	-	-	-	-
Tissue damage	-	+	+	-	+	+	+	+	-
Necrotic tissue	-	-	-	-	-	-	-	-	-
Apoptotic bodies	-	+	+	-	+	+	+	+	-
Lamina propria edema	-	++	++	-	++	++	++	++	-
Epithelial/glandular atrophy	-	+	+	-	+	+	+	+	-
Intestinal metaplasia	-	+	+	-	+	+	+	+	-
*Helicobacter pylori* (ATCC 43526) density *	-	++	++	+	++	++	++	++	+

DMSO—dimethyl sulfoxide. TT—triple therapy (amoxicillin 50.0 mg/kg, clarithromycin 25.0 mg/kg, and omeprazole 20.0 mg/kg). CHEBRP—concentrations of crude hydroalcoholic extract of Brazilian red propolis (I: 18.25 mg/kg; II: 37.5 mg/kg; III: 75.0 mg/kg; IV: 150.0 mg/kg and V: 300.0 mg/kg). ^¤^ Infected groups. * Modified Giemsa staining. ** Normal resident cells. Scored for intensity: (-) absent, (+) mild, (++) moderate, as previously described by the updated Sydney classification [27,28].

**Table 4:** Qualitative analyses of gastric tissues (antrum-pyloric) of the experimental groups during *Helicobacter pylori* (clinical isolate) infection in rats.

Histological Criteria	Uninfected	Infected	DMSO^¤^	TT^¤^	CHEBRP I^¤^	CHEBRP II^¤^	CHEBRP III^¤^	CHEBRP IV^¤^	CHEBRP V^¤^
Chronic inflammation	+ **	+++	++	+	++	++	++	++	+
Neutrophil polymorph infiltration (activity)	-	+	+	-	+	+	+	+	-
Eosinophils	-	-	-	-	-	-	-	-	-
Macrophages	-	+	+	-	+	+	+	+	-
Lymphocytes	+	+++	++	++	++	++	++	++	++
Plasma cells	+	+	+	+	+	+	+	+	+
Giant foreign body cells	-	-	-	-	-	-	-	-	-
Fibroblast	-	-	-	-	-	-	-	-	-
Tissue damage	-	++ − ulcer	+	-	+	+	+	+	-
Necrotic tissue	-	-	-	-	-	-	-	-	-
Apoptotic bodies	-	++	+	-	+	+	+	+	-
Lamina propria edema	-	++	++	-	++	++	++	++	-
Epithelial/glandular atrophy	-	++	+	-	++	++	++	++	-
Intestinal metaplasia	-	+	+	-	+	+	+	+	-
*Helicobacter pylori* (clinical isolate) density *	-	++	++	+	++	++	++	++	+

DMSO—dimethyl sulfoxide. TT—triple therapy (amoxicillin 50.0 mg/kg, clarithromycin 25.0 mg/kg, and omeprazole 20.0 mg/kg). CHEBRP—concentrations of crude hydroalcoholic extract of Brazilian red propolis (I: 18.25 mg/kg; II: 37.5 mg/kg; III: 75.0 mg/kg; IV: 150.0 mg/kg and V: 300.0 mg/kg). ^¤^ Infected groups. * Modified Giemsa staining. ** Normal resident cells. Scored for intensity: (-) absent, (+) mild, (++) moderate, (+++) severe, as previously described by the updated Sydney classification [27,28].

## Data Availability

All data generated and analyzed during this study are available from the corresponding author on reasonable request.

## Sample Availability

Samples of the compounds are not available from the authors.
